# Natural polymer composites for sustainable dust suppression: a soil mineralogy-guided chemical design

**DOI:** 10.3389/fchem.2025.1589969

**Published:** 2025-06-25

**Authors:** Dianqiu Yang, Wenhao Wang, Chenyu Zhu

**Affiliations:** School of Materials Science and Engineering, Chang’an University, Xi’an, China

**Keywords:** dust suppressant, grey correlation degree, pearson correlation, soil composition, wettability, dust reduction

## Abstract

Road construction-related dust pollution significantly endangers both environmental sustainability and public health, in contrast to traditional dust management techniques like water spraying, which are resource-demanding. The development of eco-friendly dust suppressants with tailored mineral-polymer interactions remains a critical challenge in sustainable material chemistry. This research created a novel composite dust suppressant using sodium alginate (SA), carboxymethyl cellulose (CMC-Na), hydroxypropyl trimethylammonium chloride chitosan (HTCC), gelatin (GEL), and glycerol (GLY). Through orthogonal experiments, the ideal composition was identified as SA (34.8%), CMC-Na (8.7%), HTCC (34.8%), GEL (4.3%), and GLY (17.4%). The dust suppressant demonstrated superior film-forming properties and enhanced wettability. During performance tests, the dust suppressant achieved over 99% dust reduction at a wind speed of 15 m/s across five different soil types. Analyses using Grey correlation and Pearson correlation were performed to explore the impact of soil composition. The results revealed that soil components, including Al_2_O_3_, CaO, SiO_2_, TiO_2_, and MnO, improved water retention, agglomeration rate, and wind erosion resistance. The results underscore the vital importance of soil composition in improving the effectiveness of dust suppression. The new suppressant demonstrated significantly better dust control capabilities. This renders it an exceptionally efficient method for reducing dust contamination in road building. Additionally, it provides a feasible method for safeguarding the environment during construction processes. Through cost analysis, compared with traditional water spraying methods, the overall cost is reduced by about 40%. And with a wide range of raw materials and simple preparation, it can meet the needs of large-scale production. This work elucidates the role of polymer-mineral chemistry in dust suppression, offering a scalable, green alternative that bridges environmental engineering and sustainable material science.

## Introduction

Dust suppression in construction environments remains a persistent challenge due to the limitations of conventional chemical suppressants (Wu et al., 2020). In the field of highway engineering, the high filling and low excavation of roadbed construction (Yan et al., 2022), the mixing and transportation of pavement engineering materials, the air pressure generated by vehicle driving, and the friction between tires and the ground all generate a large amount of dust. Worker engaged in construction in such dusty environments are frequently exposed to fine dust particles over extended periods, leading to the inhalation of these particles and the subsequent development of respiratory diseases, including pneumoconiosis. This disease ranks among the most critical occupational illnesses in China (Zhang F. et al., 2023). The detrimental impact of hazards extends beyond the immediate health of workers; it also impedes the growth and advancement of the national economy. Furthermore, an excess of dust in the atmosphere will impair visibility and increase the likelihood of road traffic accidents, which will in turn result in additional accidents. Extended dispersal of particulate matter in the air could endanger local residents’ health and intensify the city’s haze, posing a threat to the urban populace’s health (SLA B et al., 2022). Dust pollution is escalating in severity, with an increasing recognition of its permanent effects on human health and the environment (Li et al., 2023).

Traditional dust suppression methods such as watering and preparing single dust suppressants (Wu AM. et al., 2024; Zhang X. et al., 2023) face limitations in low water efficiency, short usage time (Wang K. et al., 2024), and environmental risks such as soil pollution (Zhao et al., 2021). This has led to a shift towards multifunctional composite dust suppressants, which synergistically integrate adhesion, wetting, and moisture retention through the physical and chemical hybridization of natural polymers (Ding et al., 2020; Wang Q. et al., 2023), achieving sustainable dust control, reducing ecological impact (Wu L. et al., 2024), and improving durability under extreme conditions (Wang X. et al., 2023).

Composite chemical dust suppressants often have the main functions of traditional dust suppressants, especially suitable for use in complex environments. Now, road dust suppressants are developing towards diversified composite and functional specialization. Zhang and Zhang, 2021 selected sodium alginate, carboxymethyl chitosan, sodium polyacrylate, and sodium dodecyl sulfate, and determined the formula of the composite dust suppressant using response surface methodology. It was found to have good anti freezing, anti-rain erosion, and anti-wind erosion properties. (Zhang et al., 2023a) employs soy protein isolate as the base material and experiences polyacrylamide chemical alterations to create a combined dust inhibitor, ensuring efficient diffusion and moistening of coal dust (Yang et al., 2024). employs substances like sodium chloride, calcium chloride, hexahydrate magnesium chloride, and polyacrylamide to inhibit dust, with research indicating their effectiveness in resisting wind erosion. (Wang et al., 2024a) employed sodium dodecyl benzene sulfonate, the nonionic surfactant polyethylene glycol pisooctyl phenyl ether, and inorganic salt sodium sulfate in the creation of dust suppressants. Dust suppressants markedly enhanced the moisture absorption efficiency of coal specimens and fostered improved engagement between the mixture and the coal’s exterior. Currently, global research and development focus more on coal chemical dust suppressants, yet there’s a noticeable scarcity of studies on road dust suppressants (Wang et al., 2023b). Presently, it remains uncertain if the efficacy of applying an identical dust suppressant varies across diverse soils. Furthermore, it remains undocumented if various soil elements influence the reduction of dust. impact of agents that inhibit dust.

In view of this, SA, CMC-Na, HTCC, GEL, and GLY were used as the main raw materials to prepare composite dust suppressant. By selecting five representative soils, the differences in soil composition were analyzed, and the reasons for the changes in neutral energy of the prepared dust suppressant in different soils were revealed from the perspective of soil composition. Research has found this dust suppressant can effectively wet and suppress dust in different soils, and has a good effect on preventing and controlling road dust. After spraying dust suppressants, the content of Al_2_O_3_, CaO, SiO_2_, TiO_2_, and MnO is strongly correlated with the wind erosion resistance, agglomeration rate, and water retention rate of different soil samples. This study creates a chemistry-based structure for developing biodegradable, soil-adaptive dust suppressants by linking mineral-specific binding processes to the efficiency of suppression. The results of our study enhance the basic knowledge of polymer-mineral chemistry and present a scalable approach to minimize environmental impact in infrastructure.

## Materials and methods

### Raw materials

#### Dust suppressant materials

SA: Purchased from Shandong Yousuo Chemical Technology Co., Ltd. Product code: YS-SA-001, analytical grade, purity ≥99%. CMC-Na: Purchased from Shandong Yousuo Chemical Technology Co., Ltd. Product code: YS-CMC-003, analytical grade, viscosity 500–800 mPa·s. HTCC: Purchased from Qianjin Chemical Co., Ltd. Product code: QJ-HTCC-05, substitution degree ≥98%, analytical purity. GEL: Purchased from Sinopharm Chemical Reagent Co., Ltd. Product code: G0041, Type B. GLY: Purchased from Sinopharm Chemical Reagent Co., Ltd. Product code: G6201, purity ≥99.5%.

#### Soil information

Selecting Inner Mongolia loess (IML), Shanxi loess (SXL), Sichuan loess (SCL), Zhejiang red soil (ZJRS), and Northeast black soil (NBS) as research objects. The selected soil covers the arid region, Loess Plateau, southwestern humid region, southeastern red soil region, and northeastern black soil region. By using soil under different climatic and geological conditions, the selected soil has geographical distribution representativeness, ensuring that the experimental results have practical engineering reference value. They were first crushed in a universal high-speed ball mill, and then screened to obtain coal powder with a particle size of about 100 mesh. Then, the screened soil samples were dried in a vacuum oven at 60°C for 24 h, taken out and allowed to cool, and preserved in airtight plastic bags for later experimental trials.

### Preparation of composite dust suppressant

The preparation protocol was adapted from established methods for natural polymer composites (Zhang and Zhang, 2021), with optimization based on orthogonal experimental design. Factors affecting orthogonal experiments encompass the quality of SA, CMC-Na, HTCC, and GEL. Take the settling time and wind erosion rate of the dust suppressant as key evaluation indicators, and then use the range method to analyze and determine the optimal formula for the dust suppressant.

Place a 250 mL three-necked flask into a water bath, set the temperature to 80°C, gradually and evenly introduce and distribute GEL, then raise the temperature to 60°C, introduce SA and CMC-Na while stirring, and continue stirring for a specific pe After a certain duration, introduce HTCC, following the addition of GLY, mix in water to achieve a solution volume of 100 mL. The solution was mixed thoroughly for 1 h to obtain a homogeneously dispersed and stabilized solution.

### Experimental methods

#### Settlement time test

The wetting performance of dust suppressants on different soil samples was studied through settling time testing. It was performed following a modified protocol based on ASTM D422-63 (ASTM International, 2022). 25mL dust suppressant solution was prepared in a beaker and 0.1 g of soil particles were poured onto the surface of the solution. Automatically record the settling time, which is the time it takes for the last soil sample particle to settle from the surface of the solution.

#### Viscosity test

The viscosity of the dust suppressant signifies its capacity to adhere and cluster dust particles, serving as a crucial measure of its effectiveness. The viscosity test was conducted according to ASTM D2196-18 (ASTM International, 2018). The viscosity of the dust suppression solution was measured at 25°C using a Brookfield DV2T rotational viscometer (Brookfield Engineering Laboratories, Massachusetts, United States).

#### Wind erosion performance test

After passing the dust sample through a sieve with 100 mesh, it was dried to a uniform weight prior to its transfer into a culture dish. The optimal ratio of dust suppressant solution was sprayed at a rate of 2L/m. After the dust sample was dried, a blower was used to continuously blow air at a wind speed of 15 m/s for 30 min to measure the wind erosion rate. The calculation of wind erosion rate is shown in formula 1:

$$\alpha =\frac{M_{0}-M_{1}}{m}\times 100\%$$ (1)

Where Q: wind erosion rate, %; M_1_: the mass of the culture dish after blowing, g; M_0_: the mass of the culture dish before blowing, g; m: the mass of the soil sample, g.

#### Water retention rate test

The dust suppressant plays a crucial role in water retention, creating a solidified layer on the soil sample’s surface and lessening water evaporation (Zhang et al., 2018). Firstly, 20 g sample of soil was measured and evenly distributed in a Petri dish via a sieve. The dust suppressant solution was evenly dripped on the surface of the soil sample. The water retention property of the solution was assessed by recording the weight loss of the sample (Wu et al., 2025).

#### Agglomeration rate test

An appropriate quantity of the soil sample should be placed in the culture dish. The prepared solution should then be sprayed evenly onto the soil sample (Huang et al., 2021). After complete penetration, place it in a drying oven at 110°C for 2 h to obtain a mass M_0_. Use a 40 mesh sieve to screen the sample, and weigh the mass on the 40 mesh sieve as m. The calculation of agglomeration rate is shown in formula 2:

$$Q=\frac{M_{0}‐M_{1}}{m}\times 100\%$$ (2)

Where Q is the agglomeration rate, %; M_1_ is the mass of the culture dish after blowing, g; M_0_ is the mass of the culture dish before blowing, g; m is the mass of the soil sample, g.

#### Contact angle test

The contact angle between the solution and the soil specimen serves as a measure to evaluate the solution’s capacity to wet the dust sample (Zhang et al., 2022), and the ability of the solution to moisten the dust serves as a crucial indicator influencing the effect of dust suppression (Zhang F. et al., 2023). Consequently, a contact angle tester was employed to determine the contact angle between the dust suppressant solution and the dust sample, and to assess the variance in wettability between them (Yu et al., 2022).

#### XRD analysis

Various soil specimens were placed in a vacuum drying chamber at 60°C to allow drying until the mass reached a stable state. Next, the desiccated substance was pulverized in a mortar to extract target particles for the XRD analysis. Samples that were prepped were positioned in the A polycrystalline X-ray diffractometer utilized in testing. Ultimately, the Jade 6.0 software was utilized for the analysis of the acquired graphs.

#### XRF analysis

Grind different soil samples and press it into thin sheets using a tablet press. The chemical composition of the sample was determined using an X-ray fluorescence spectrometer.

#### Grey correlation analysis

Grey correlation analysis quantitatively assesses a system’s dynamics to determine the extent of correlation among its various factors (Li et al., 2023), the fundamental concept revolves around ascertaining the extent of correlation among the elements, contingent on their similarity level (Gong et al., 2020). In this paper, grey correlation is used to assess the effect of single minerals on wind erosion resistance, agglomeration rate and water retention of soil samples.

#### Pearson correlation

In the field of natural sciences, Pearson correlation coefficient, a commonly used statistical metric, is utilized to determine the intensity of the linear correlation between two variables (Cheng et al., 2022; Zhao et al., 2023). Its applications are numerous and include the examination of complex datasets comprising multiple factors (Xu et al., 2022). This paper examines the effects of dust suppressants on soil wind erosion resistance performance, agglomeration rate, and water retention rate from the perspectives of minerals and soil.

#### Anti water erosion experiment

The waterproof and long-term stability of dust suppressants are key parameters for measuring their performance. Evenly spread the dried dust sample to a constant weight in a culture dish, spray the dust suppressant solution at a rate of 2 L/m^2^. After the surface forms a solidified layer, it was placed in a UV aging chamber. The conversion relationship between outdoor sunlight exposure and UV radiation time in the chamber is shown in Table 1. After 4 days of UV aging, tilt the culture dish at 30° and fix it. Using an indoor sprinkler system to simulate natural rainfall, with a controlled flow rate of 4 mL/(s·m^2^), spray continuously for 10 min at a height of 0.5 m directly above the culture dish. After the spraying is completed, dry the sample to a constant weight and weigh the mass, and repeat the simulated rainfall experiment five times. The water erosion rate (β) is calculated according to formula 3:

$$\beta =\frac{m_{0}‐m_{n}}{M}\cdot 100\%$$ (3)

**TABLE 1:** Conversion table of sunlight and indoor UV radiation time.

Condition	Time/d				
Sunlight	120	240	480	720	840
UV Radiation	1	2	4	6	7

Where m_0_ and m_n_ are the mass of the culture dish before and after rainfall, g; M are the initial mass of the soil, respectively, g.

## Results and discussion

### Orthogonal test

The orthogonal optimization employed a systematic L_9_ (3^4^) matrix to evaluate four critical components of the dust suppressant: SA (1%, 1.5%, 2%), CMC-Na (0.5%, 1.0%,1.5%), HTCC(1%, 1.5%, 2%), and GEL (0.25%,0.5%,0.75%). Key responses, including settling time and wind erosion rate, were measured to identify the optimal formulation.

**FIGURE 1:**
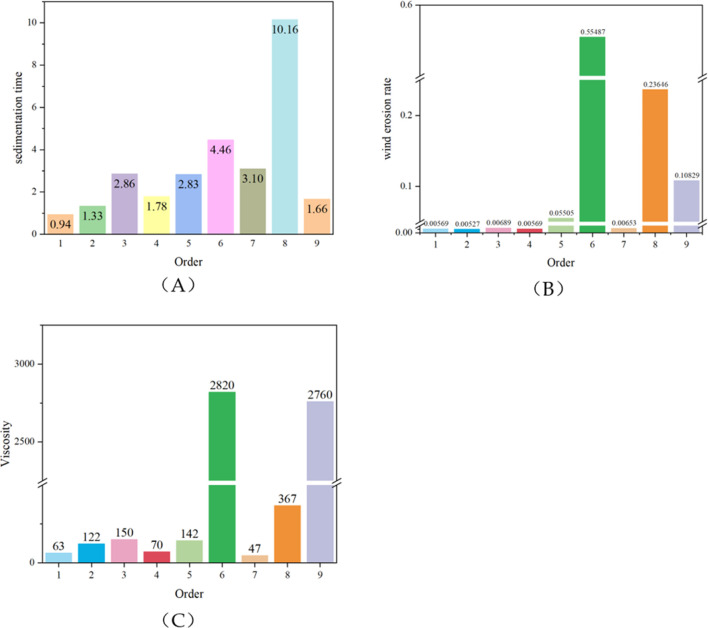
Orthogonal analysis result **(A)** sedimentation time **(B)** wind erosion rate **(C)** Viscosity.

Determine the optimal concentration level based on K obtained from orthogonal experiments, and determine the optimal component factors based on the range (R). According to the principle that a smaller settling time is better, a smaller K represents a faster settling time, and the dust suppressant has better wetting properties for the soil. Calculate based on the results of Figure 1A, 1.71 for K_11_(A), 1.94 for K_11_(B), 1.59 for K_12_(C), 1.81 for K_11_(D), levels 1, 1, 2, and one are the optimal levels for components SA, CMC-Na, HTCC, and GEL, respectively. Then, Maximum 3.60 by R_C_, R_C_ > R_A_ > R_D_ > R_B_ indicates that HTCC is the most important component for improving the wetting properties of the dust suppressant, followed by SA, GEL, and CMC-Na. Since the smaller the wind erosion loss rate, the better the dust suppression effect. Calculate based on the results of Figure 1B, 0.01 for K_21_(A), 0.01 for K_21_(B), 0.02 for K_23_(C), 0.06 for K_21_(D), levels 1, 1, 3, and one are the optimal levels for component factors SA, CMC-Na, HTCC, and GEL, respectively. Furthermore, Maximum 0.24 by R_C_, R_C_ > R_B_ > R_A_ > R_D_ indicates that HTCC is the most important component factor controlling wind erosion rate and has the most significant dust suppression effect, followed by CMC-Na, SA, and GEL. Calculate based on the results of Figure 1C, 1058.07 for K_33_(A), 1910.00 for K_33_(B), 1083.33for K_31_(C), 996.40 for K_32_(D), levels 3, 3, 1, and two are the optimal levels for component factors SA, CMC-Na, HTCC, and GEL, respectively. Then, Maximum 1849.93 by R_B_, from R_B_ > R_C_ > R_A_ > R_D_, it can be concluded that CMC-Na is the most important component factor in controlling viscosity, followed by HTCC, SA, and GEL. However, when the viscosity is too high, it is extremely easy to crack after film formation and drying. Therefore, considering the actual use situation, the optimal level should have good operability while ensuring a certain effect. Therefore, the optimal combination is levels 3, 1, 3, and 1, that is, SA is 2%, CMC-Na is 0.5%, HTCC is 2%, GEL is 0.25% and GLY is 1%. SA, CMC-Na, HTCC, and GEL GLY account for 34.8%, 8.7%, 34.8%, 4.3%, and 17.4% of the total mass, respectively. Experimental verification was conducted on the final determined level, and the film-forming property was good. The surface of the dust sample was continuous and intact as a whole, without any small cracks.

### Soil XRD analysis

**FIGURE 2:**
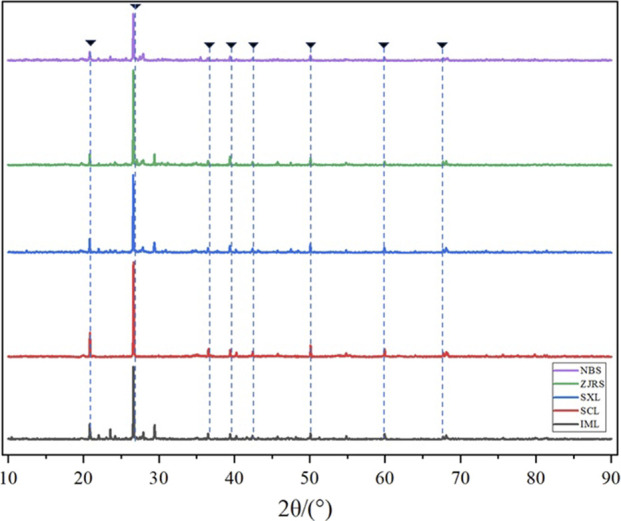
Soil XRD analysis.

According to the XRD analysis of different soils in Figure 2, the waveforms of the five soils overlap in some places. The main component of the five types of soil is quartz, and the XRD diffraction peaks of quartz are marked in the figure. The Qualitative analysis was conducted on the XRD diffraction pattern, and quantitative analysis was performed using the RIR method. It was found that the quartz content of NBL was 65.0%. At the same time, it was found that the quartz content in SXL, SCL, ZJRS, and IML was 85.5%, 95.3%, 70.7%, and 67.7%, respectively. The main secondary minerals are clay minerals such as kaolinite, montmorillonite, and calcite. Montmorillonite, due to its layered structure, may enhance the adsorption of dust suppressants on soil particles; The rigid structure of kaolinite may affect the strength of the solidified layer of dust suppressants. The proportion of mineral composition in soils from different regions varies significantly.

### Dust suppression experiment

#### Water retention rate experiment

The final water retention rates after 12 h varied significantly among the soil samples, with IML retaining the highest percentage (83.07%), followed by SXL (82.02%); SCL(80.01%); NBS(78.38%) and ZJRS exhibiting the lowest retention rate (76.78%). the overall water retention rates from high to low were: IML > SXL > SCL > NBS > ZJRS. The difference in its water retention rate is mainly due to the difference in the binding energy between water and soil, which leads to changes in its water retention rate. Dust suppressants can partially remain in the dust gaps during the infiltration process, forming a solid layer with high adhesion to firmly adsorb dust and minimize the water depletion of the dust specimen. The structure of the soil sample exhibits a particular hydrophobic quality, which facilitates the aggregation of water molecules. After water evaporated, the top layer of the soil sample exhibits a loosening effect (Huang et al., 2021) The network structure demonstrates potent binding energies for water molecules, impeding their movement.

The final water retention rates after 12 h varied markedly among mineral components, with MnO exhibiting the highest retention (91.01%), followed by SiO_2_ (89.07%), TiO_2_ (88.83%), and Fe_2_O_3_ (87.92%), while Na_2_SiO_3_ showed the lowest retention (77.24%), highlighting distinct hydration stability across compositions. After applying the dust suppressant to the mineral surfaces, the solution can easily infiltrate the spaces separating the particles (Yu et al., 2022). On the surface of a mineral, the primary components of the sprayed water include surface adhesion water and capillary water. Following this, the surface moisture of the minerals slowly dissipates as heat is absorbed from the air around them, leading to a persistent reduction in moisture content (Wu et al., 2020). Simultaneously, with the increase in the system’s relative humidity, there’s a heightened escape rate of water molecules from the interface to the gas phase, leading to an ongoing widening of the concentration gap between the interface and the gas phase. At the same time, based on the XRF analysis results, refer to Table 2, the content of Na_2_SiO_3_ in IML is the lowest. Due to the negative impact of Na_2_SiO_3_ on water retention rate, the water retention rate of IML is relatively good compared to other soils. ZJRS has a 2.31% higher Na_2_SiO_3_ content than IML, and the highest Na_2_SiO_3_ content in ZJRS. The content of montmorillonite in ZJRS is relatively low. Due to the ability of montmorillonite to adsorb surrounding water, its low content leads to easy evaporation of water in ZJRS, reducing its water retention capacity.

**TABLE 2:** Soil XRF analysis.

Soil	SiO_2_	TiO_2_	Al_2_O_3_	Fe_2_O_3_	MnO	MgO	CaO	Na_2_O	K_2_O	LOI	Total
	w(M)/10^−2^										
SXL	60.71	0.60	11.34	3.78	0.07	2.44	8.31	1.60	2.30	9.52	100.67
SCL	67.99	1.12	13.63	7.58	0.04	0.63	0.56	2.12	1.29	5.64	100.60
IML	65.35	0.66	11.64	2.79	0.06	1.42	5.92	0.05	2.76	9.98	100.63
ZJRS	64.48	0.62	12.47	3.46	0.09	1.51	4.73	2.36	2.32	8.12	100.16
NBS	51.64	0.55	10.46	3.32	0.07	1.09	2.30	1.96	2.13	27.12	100.64

#### Agglomeration rate experiment

The agglomeration rates of soil samples sprayed with dust suppressants vary greatly. Among them, SCL and ZJRS have the best agglomeration rates, which are 66.512% and 70.891%, respectively. IML, SXL, and NBS have relatively poor agglomeration rates, which are 10.09%, 14.73%, and 12.82%, respectively. The main reason for its different agglomeration rates is due to the difference in adhesion energy between dust suppressants and different soils, which leads to changes in its agglomeration rate. ZJRS and SCL have a higher agglomeration rate due to their good adhesion with dust suppressants, which can bind fine dust particles together and suppress their dust emission. The final agglomeration rates exhibited marked variations across mineral compositions, with Na_2_SiO_3_ demonstrating the highest rate (99.64%), followed by K_2_SiO_3_(97.08%), MgO(92.15%), Fe_2_O_3_(90.53%), SiO_2_(74.18%), TiO_2_(68.66%), MnO(46.05%), CaO(28.01%), and Al_2_O_3_(23.28%). This is mainly due to the differences in adhesion energy between different individual minerals. Dust suppressants use their own viscosity to aggregate small dust particles after spraying, forming larger particles that can effectively resist lifting caused by external interference or induced airflow. Based on the results of soil XRF analysis, due to the highest content of Na_2_SiO_3_ in ZJRS and SCL, the agglomeration rate of ZJRS is relatively good. The content of Na_2_SiO_3_ in IML is the lowest, so the agglomeration rate of IML is poor. The order of Na_2_SiO_3_ content in the five soils is ZJRS > SCL > NBS > SXL > IML, which is roughly the same as the soil agglomeration rate.

#### Contact angle test

**FIGURE 3:**
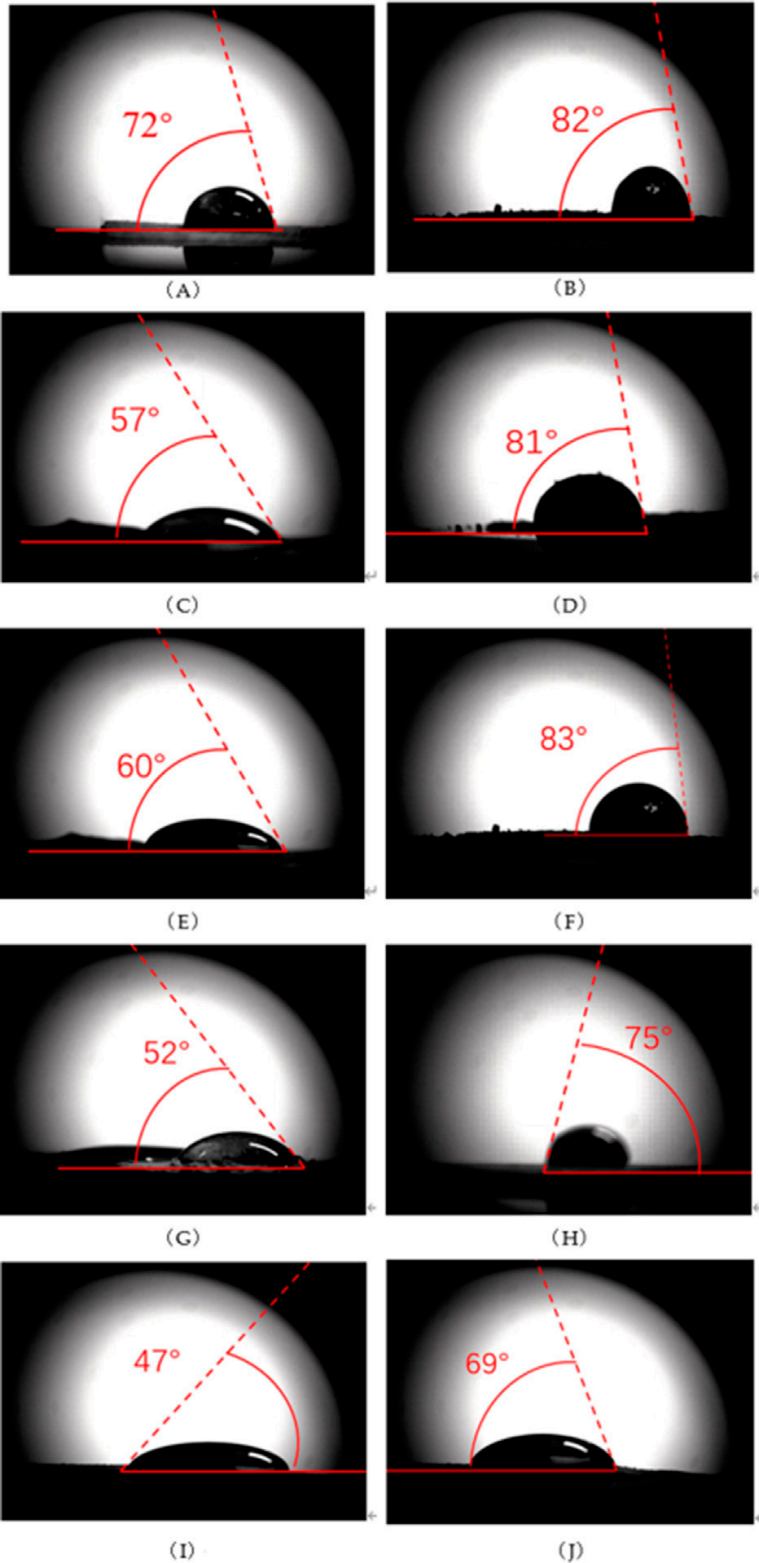
Soil contact angle **(A)** Dust suppressants and NBS **(B)** Water and NBS **(C)** Dust suppressants and IML **(D)** Water and IML **(E)** Dust suppressants and SXL **(F)** Water and SXL **(G)** Dust suppressants and SCL **(H)** Water and SCL **(I)** Water and ZJRS **(J)** Dust suppressants and ZJRS.

The contact angles before and after spraying dust suppressants on different soils are shown in Figure 3. The contact angles of water and NBS, IML, SCL, SXL, and ZJRS are 82°, 81°, 83°, 75°, and 69°, respectively, which are close to the critical value of non-wetting 90°. This indicates that the hydrophilicity of these soil samples is poor and it is difficult to be wetted by water. The dust suppression efficiency is low by simply spraying water. After using dust suppressants, The contact angle between dust suppressants and soil samples is reduced compared to that between water and soil. The contact angles of dust suppressants and NBS, IML, SCL, SXL, and ZJRS are 72°, 57°, respectively. 60°, 52°, 47°. This indicates that the prepared dust suppressant has good wetting properties.

#### Wind erosion performance

The wind erosion resistance rates of the tested soils were as follows: IML (99.46%), SCL(99.03%), SXL (99.64%), ZJRS (99.66%), and NBS(99.01%). Five different types of soil have dust suppression rates exceeding 99% and perform well when the wind speed of the small wind tunnel reaches 15 m/s. The wind erosion resistance rates of the minerals were determined as follows: MgO(97.57%), MnO (99.45%), Fe_2_O_3_(98.49), CaO(89.02%), SiO_2_(99.02%), TiO_2_(98.37%), Al_2_O_3_(99.64%), Na_2_SiO_3_ (99.95%), and K_2_SiO_3_(99.77%). This is mainly because after drying, a solidified layer will form on the mineral surface after spraying dust suppressants, which can effectively cover the surface of the dust sample and prevent secondary dust (Wang X. et al., 2023). Calcium oxide has relatively poor wind erosion resistance, mainly due to its rapid heat release and chemical reaction with dust suppressants, which generates calcium hydroxide and destroys its solidified layer. Due to the rupture of the solidified layer, its wind erosion resistance performance is poor.

**FIGURE 4:**
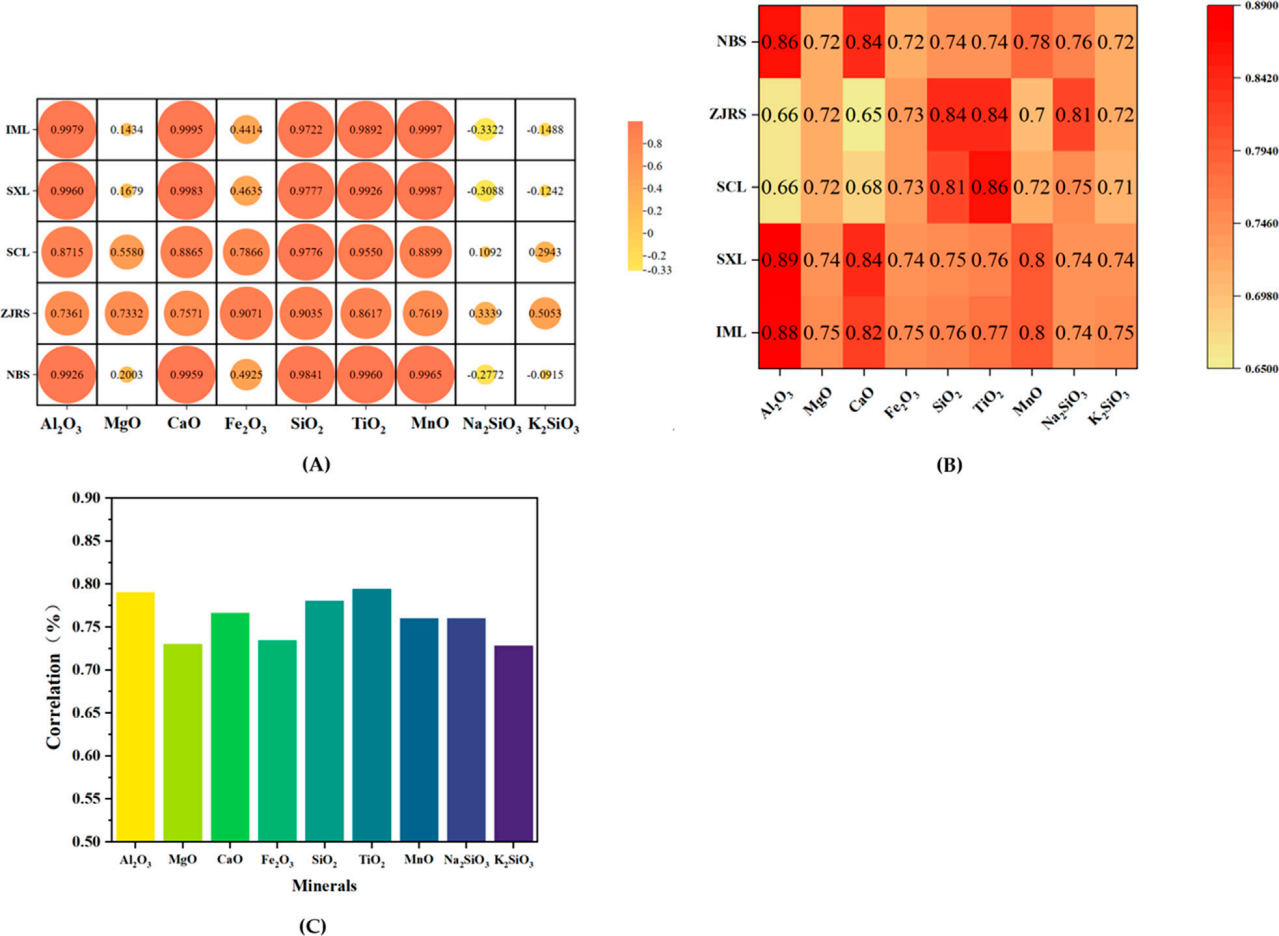
**(A)** Pearson correlation **(B)** Grey correlation analysis **(C)** Grey correlation coefficient.

According to the correlation between various indicators of minerals and different soil dust suppression indicators, Pearson correlation coefficient analysis was conducted between different minerals and five types of soil. The results are shown in Figure 4A. After spraying dust suppressants, the contents of Al_2_O_3_, MgO, CaO, Fe_2_O_3_, SiO_2_, TiO_2_ and MnO show a positive correlation with the wind erosion resistance, agglomeration rate, and water retention rate of soil samples. That is to say, the higher the content of Al_2_O_3_, MgO, CaO, Fe_2_O_3_, SiO_2_, TiO_2_ and MnO in the soil, the better the wind erosion resistance rate, agglomeration rate, and water retention performance of soil samples sprayed with dust suppressants. Among them, the content of Al_2_O_3_, CaO, SiO_2_, TiO_2_, and MnO is strongly correlated with the wind erosion resistance, agglomeration rate, and water retention rate of different soil samples after spraying dust suppressants. The chemical composition of soil reflects the types of minerals in the soil. SiO_2_ mainly exists in the form of quartz, while Al_2_O_3_, CaO, TiO_2_, and MnO are usually associated with clay minerals such as kaolinite or montmorillonite (Bai et al., 2010). Due to its layered structure, clay minerals can effectively enhance the adsorption of dust suppressants on dust surfaces. Therefore, the physical and chemical properties of different minerals in soil can affect the dust suppression performance of dust suppressants. Using the grey relational theory, this study examines the effects of dust suppressants on soil wind erosion resistance performance, agglomeration rate, and water retention rate from the perspectives of minerals and soil. The coefficients of grey correlation analysis are shown in Figure 4B.

On the basis of obtaining the grey correlation coefficient above, and then substituting it into the formula, the impact of minerals in soil on the wind erosion resistance performance, agglomeration rate, and water retention rate of soil after spraying dust suppressants can be analyzed in Figure 4C.

According to the results of grey correlation degree, TiO_2_ possesses the greatest effect on the soil samples. The relationship between the correlation degree is Ti0_2_>Al_2_O_3_>SiO_2_>CaO > MnO = Na_2_SiO_3_>Fe_2_O_3_>MgO > K_2_SiO_3_, which is consistent with the Pearson correlation coefficient. After spraying dust suppressants, the contents of Al_2_O_3_, CaO, SiO_2_, TiO_2_, and MnO in the soil can effectively improve the soil sample’s wind erosion resistance, agglomeration rate, and water retention rate. Since the contents of Al_2_O_3_, CaO, SiO_2_, and TiO_2_ in NBS are all less compared to other soils, its resistance to wind erosion is slightly lower than the other four types of soil. By combining XRD analysis, it was found that Al_2_O_3_ mainly originates from kaolinite, and its structure can enhance the binding effect between dust suppressants and particles. By combining soil mineral composition analysis, it is possible to better link soil composition and performance.

#### Water erosion resistance performance

**FIGURE 5:**
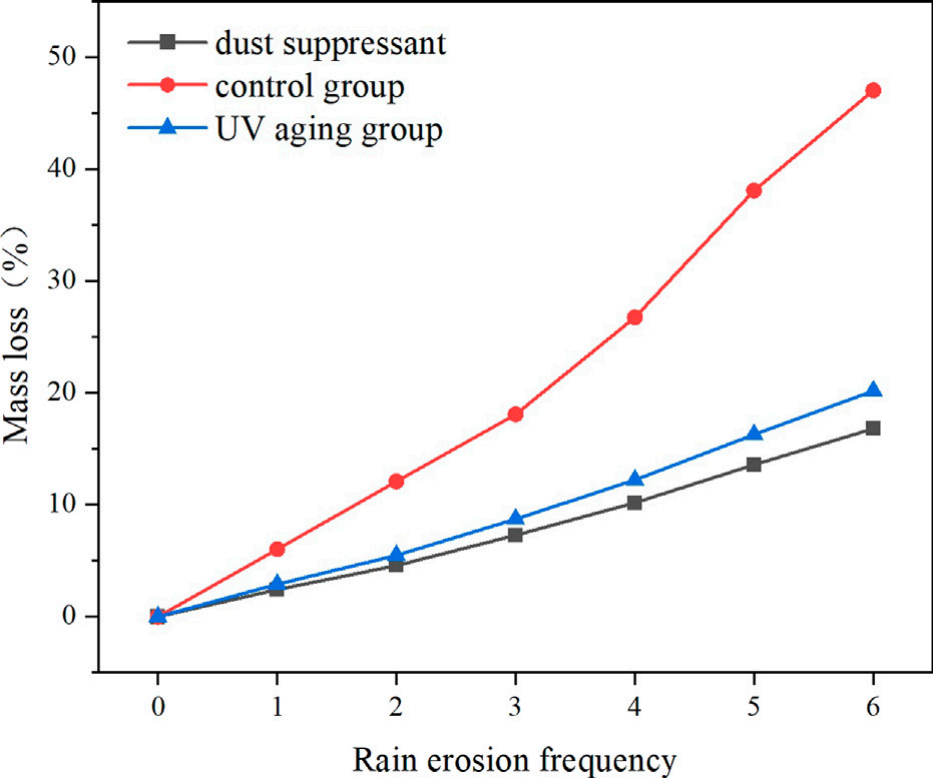
Changes in rain erosion rate.

As shown in Figure 5, the rain erosion rate of the non-UV aging dust suppressant slowly increased with the increase of rainfall frequency, but its value remained significantly lower than that of the control group with clean water. During the first water erosion, the rain erosion rate of the dust suppressant was 2.42%. By the sixth rainfall, the erosion rate of the dust suppressant increased to 16.81%, while the clear water group reached as high as 47.04%. This result indicates that the dust suppressant effectively inhibits rainwater infiltration and particle dispersion, enhancing the bonding strength between particles. Disperse stress and reduce crack propagation through van der Waals forces and hydrogen bonding interactions.

After four consecutive days of UV irradiation, the anti-rain erosion performance of the dust suppressant showed a slight decrease. After the sixth water erosion, the water erosion rate increased from 16.81% to 20.17% after UV aging, demonstrating excellent resistance to rain erosion. Dust suppressants can effectively maintain the integrity of the solidified layer and have good durability. Compared with the traditional water spraying method, although it requires a certain raw material cost, its good dust suppression performance greatly reduces the number of spray times. The effect of spraying water is relatively short, making it difficult to maintain the dust suppression effect for a long time. It is prone to failure due to rainwater erosion and requires frequent re spraying, which may result in higher long-term costs. Meanwhile, by using biodegradable materials, the risk of soil pollution caused by traditional chemical dust suppressants is avoided.

## Conclusion

This study demonstrates the rational design of a ternary alginate chitosan composite material for sustainable dust suppression by utilizing the chemical interactions between natural polymers and soil minerals. The main conclusions are as follows:

Through orthogonal experimental design, the interaction between various factors was analyzed, and the mass fractions of SA, CMC-Na, HTCC, GEL, and GLY were found to be 34.8%, 8.7%, 34.8%, 4.3%, and 17.4% respectively, the film-forming property was good. The surface of the dust sample was continuous and intact as a whole, without any small cracks.

Through performance testing of dust suppressants, it was found that they have good wetting properties. At the same time, by spraying dust suppressants on different soil samples for testing, it was found that their dust suppression rates are all above 99.0%, indicating excellent resistance to wind erosion. The anti-rain erosion experiment shows that the dust suppressant maintains excellent performance under harsh conditions, and after six rain erosion cycles, it can still maintain good dust suppression performance.

Through the analysis of grey correlation and Pearson correlation coefficient, the contents of Al_2_O_3_, CaO, SiO_2_, TiO_2_, and MnO in the soil can effectively improve the soil sample’s wind erosion resistance, agglomeration rate and water retention rate. By combining clay minerals, quartz and other minerals, clay minerals promote the adhesion of dust suppressants through surface adsorption. Future research needs to further correlate mineralogical characteristics with dust suppression performance and optimize different soil adaptations. Due to the lower content of Al_2_O_3_, CaO, SiO_2_, and TiO2 in NBS compared to other soils, its resistance to wind erosion, water retention rate, and clumping rate are relatively poor.

Through cost analysis, compared with traditional water spraying methods, the comprehensive cost of spraying dust suppressants is reduced by about 40%. Compared with commercially available single component dust suppressants, the overall cost is significantly reduced. This research contributes to a deeper comprehension of how polymer-mineral chemistry is used in environmental contexts. The study merges large-scale material advancements with tangible sustainability issues, providing a guide for the creation of eco-friendly composites in worldwide infrastructure.

## Data Availability

The original contributions presented in the study are included in the article/supplementary material, further inquiries can be directed to the corresponding author.
